# Self‐healing and shape‐shifting polymers controlled by dynamic bonds

**DOI:** 10.1002/smo.20220009

**Published:** 2023-09-21

**Authors:** Shang‐Wu Zhou, Chengyuan Yu, Meng Chen, Chen‐Yu Shi, Ruirui Gu, Da‐Hui Qu

**Affiliations:** ^1^ Key Laboratory for Advanced Materials and Joint International Research Laboratory of Precision Chemistry and Molecular Engineering Feringa Nobel Prize Scientist Joint Research Center Frontiers Science Center for Materiobiology and Dynamic Chemistry Institute of Fine Chemicals School of Chemistry and Molecular Engineering East China University of Science and Technology Shanghai China

**Keywords:** dynamers, dynamic bonds, self‐healing, shape‐shifting, supramolecular chemistry

## Abstract

Dynamic chemistry refers to a type of fundamental science that involves precise construction or regulation of reactional, motional, or constitutional dynamics of chemical systems. Under the meticulous design of chemists, the nanoscopic dynamics, either molecular or supramolecular, are managed to scale up to macroscopic dynamic properties. For example, the stimuli‐induced conformational or configurational changes of polymer skeletons result in unexpected functions of polymers, such as self‐healing and shape‐shifting behaviors. This review focuses on how the microscopic dynamics of these molecular components initiate the reversible macroscopic deformation of the corresponding polymer materials upon external stimuli. The self‐healing and shape‐shifting materials are discussed in terms of the subtle molecular design, dynamic reversible mechanisms, and critical roles of the dynamic components in building these materials. Furthermore, this review puts forward the challenges and opportunities for the field of dynamic polymers in both aspects of fundamental chemistry and material fabrication. We hope this review can provide new inspiration for the development of this particular research field.

## Graphical abstract

This review focuses on how the dynamic bonds are used to drive the macroscopic structure transformation, for example, self‐healing and shape shifting, of the dynamic multi‐functional materials.

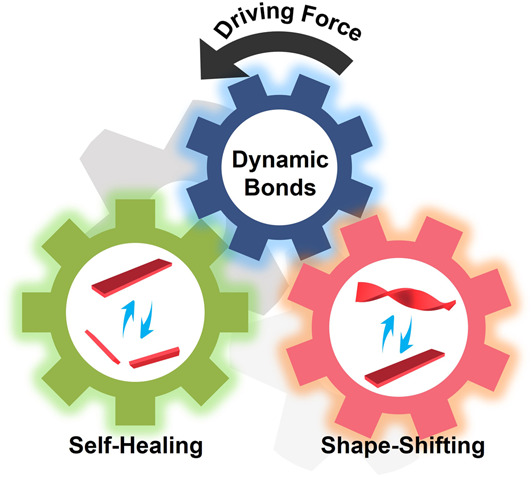

## 1 INTRODUCTION

Jean‐Marie Lehn first proposed the concept of “dynamers”,[^1^, ^2^, ^3^] which came out of his studies on supramolecular polymers and covalent dynamic polymers.[^4^, ^5^, ^6^] The dynamic assembly and disassembly of the molecules or polymers can be realized under specific conditions. Over the recent 2 decades, the field of dynamic chemistry has been extensively studied and redefined from the microstructure of individual molecules to the macro‐morphology of the resultant polymers. In‐depth studies of these micro‐molecular motifs have significantly promoted the development of dynamic polymeric materials.[^7^, ^8^, ^9^] The emergence of dynamic chemical species is a carnival of the design of new functional materials. Bowman and co‐workers proposed the concept of covalent adaptable networks (CANs).^[10]^ After embedded in dynamic covalent bonds, the original inert polymer network can be activated and become reversible under specific stimulus conditions by allowing the exchange process of its constituent dynamic linkages. Based on the concept of CANs, Leibler and co‐workers found that when the polymer backbones introduced dynamic ester bonds,^[11]^ the thermosets could be healed by reprocessing, similar to the thermoplastics. The term “vitrimer”, a particular type of CAN, often refers to the reprocessable thermosets with associative bond exchange mechanisms.

Supramolecular polymers are defined as polymeric arrays of monomeric units that are connected by reversible and highly directional noncovalent interactions, exhibiting polymeric properties in solution and bulk.[^12^, ^13^, ^14^] The adjacent monomeric units are held together by hydrogen bonding, ionic interactions, metal coordination, π‐π stacking, or hydrophobic interaction. Due to these noncovalent interactions, supramolecular polymers often exhibit novel functions such as reversibility and stimuli‐responsiveness. This paves the way for designing and constructing advanced materials with unique properties.

The fundamental dynamic bond exchange principle facilitates progress in the development of recycling and self‐healing polymers. The interface between thermoplastics and thermosets has thus been blurred by the insertion of dynamic bonds. When the dynamic bonds in the polymer network are activated by external stimuli (such as light,[^15^, ^16^, ^17^] heat,[^18^, ^19^, ^20^] pH,[^21^, ^22^, ^23^] etc.) for dynamic exchange reactions, the subsequent microstructure changes of the polymer network tend to induce a distinct shape‐shifting responsiveness in bulk. This can be explained as when the polymer network is activated, the force used to balance the polymer network is broken, and the heterogeneity of the network then releases the internal stress resulting from the network disruption, which drives the transition of macroscopic configuration of the polymer. Such stimuli‐responsive materials can be used to mimic specific natural biological motions, for example, Mimosa leaves fold to avoid harm from physical contact[^24^, ^25^]; When prey touches a flytrap, the latter will close its leaves to trap the prey.^[26]^ The imitation of such stimuli‐responsive and shape‐shifting materials is essential for the manufacture of intelligent actuators.

**FIGURE 1:**
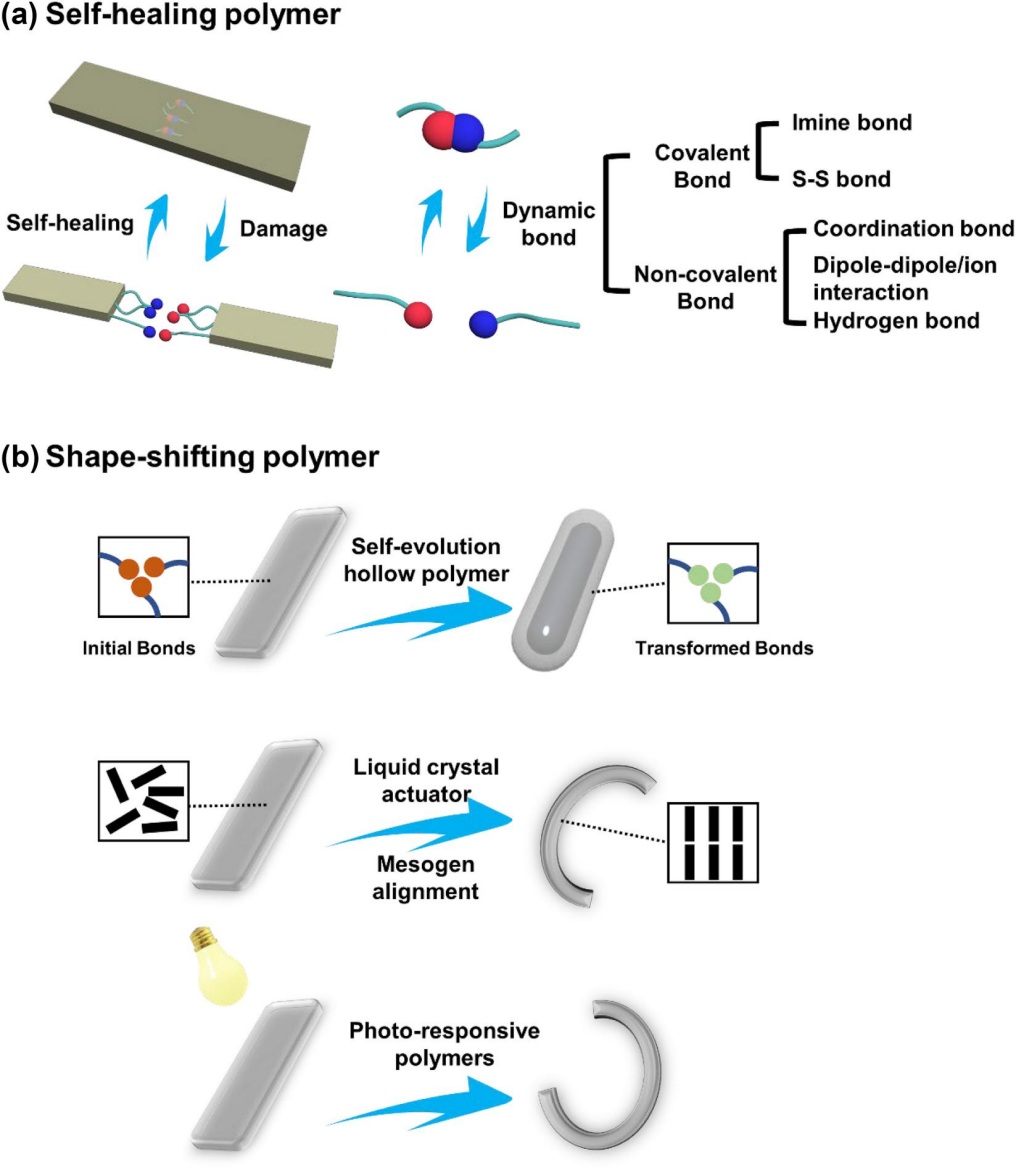
(a) Schematic illustrations of dynamic bond building blocks(S‐S bond, hydrogen bond, dipole‐dipole/ion, coordination bond, and imine bond) for self‐healing polymer. (b) Schematic illustrations of shape‐shifting polymer for self‐evolution hollow polymer, liquid crystal (LC) actuator, and photo‐responsive polymers.

The saying “*See through the appearance to perceive the essence*” applies to the field of dynamic chemistry, where the dynamic bonds and molecules are the seeds that give rise to macroscopic dynamic behaviors. The self‐healing[^3^, ^27^, ^28^, ^29^] and shape‐shifting[^30^, ^31^, ^32^] materials have been extensively discussed in recent reviews, covering their general aspects in depth. In this review, we mainly focus on the discussion of how dynamic components operate in the process of self‐healing (Figure 1a) and shape‐shifting transformation of the materials, especially self‐evolution hollow polymers and stimuli‐responsive actuators (Figure 1b), and explain the macroscopic phenomena from a micro‐molecular point of view with a detailed mechanism. Towards the end of the review, the challenges and opportunities that still exist in this research field are briefly described, with the expectation of cultivating new ideas for designing advanced dynamic intelligent materials.

## 2 SELF‐HEALING MATERIALS

The self‐healing phenomenon widely exists in nature, and various organisms maintain their physical integrity through self‐healing to maintain their basic vital signs. Inspired by nature, various dynamic bonds and molecules are structured in polymer networks[^33^, ^34^, ^35^, ^36^, ^37^] to guide self‐healing behaviors. When damaged materials exchange their internal dynamic components at the wound interface, they can be self‐healed to the original shapes and restore their initial properties. In the self‐healing progress, polymers crosslinked by dynamic covalent bonds are often accompanied by the breakage of old bonds and the formation of new bonds, and this process requires energy input (light, heat, magnetic and electric fields, and so on) to step over the energy barrier and form new dynamic covalent bonds. Like covalent bonds, polymers crosslinked by dynamic non‐covalent bonds typically involve the breaking of old bonds then forming new bonds, and the energy input helps the material to achieve faster self‐healing. In this part, some typical dynamic covalent/noncovalent bonds are discussed in detail to illustrate the mechanism of self‐healing motifs (Table 1).

**TABLE 1:** The dynamic covalent bonds and dynamic non‐covalent bonds for constructing self‐healing materials.

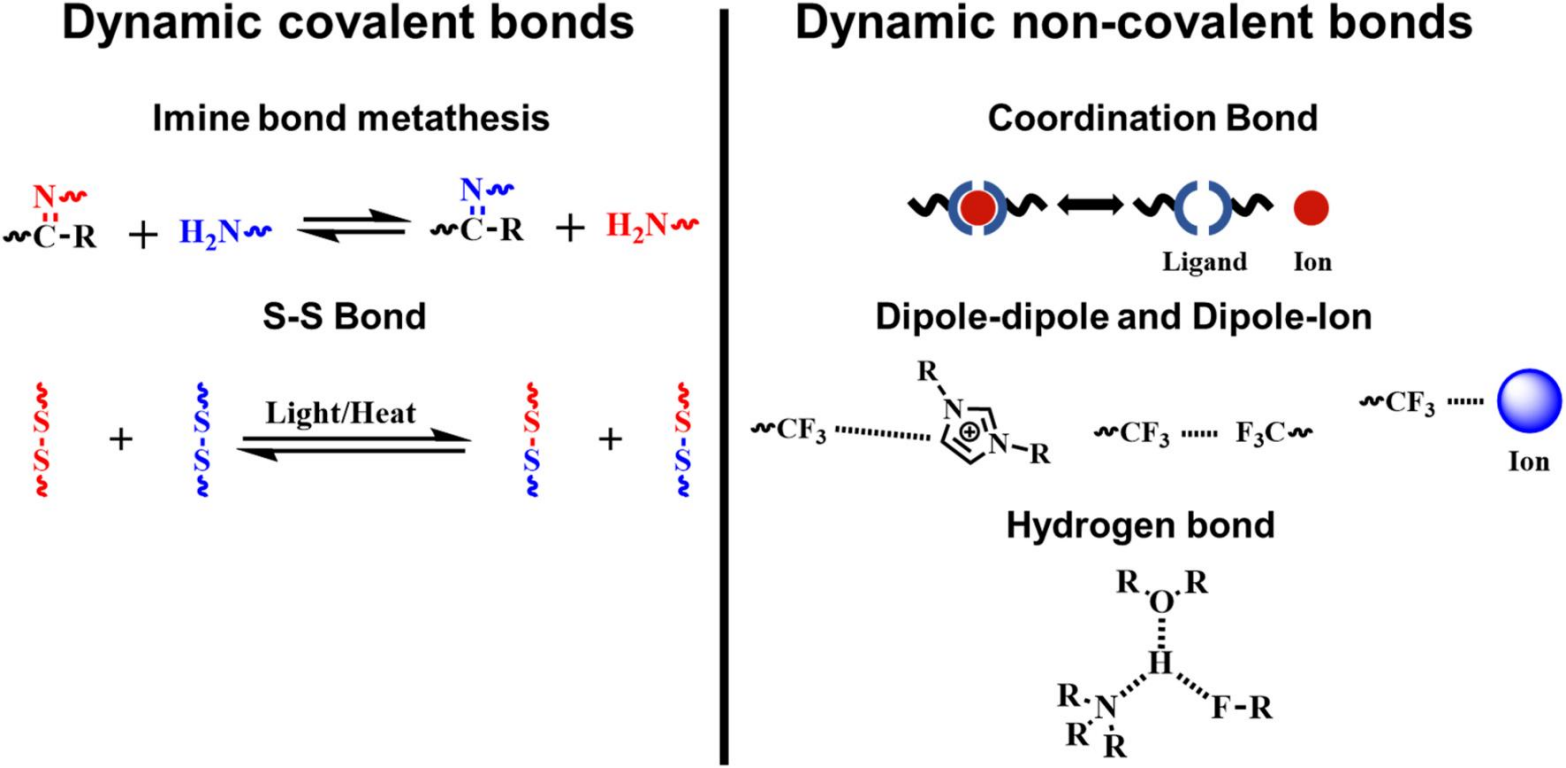

### 2.1 Dynamic covalent bonds

#### 2.1.1 Imine bond

**FIGURE 2:**
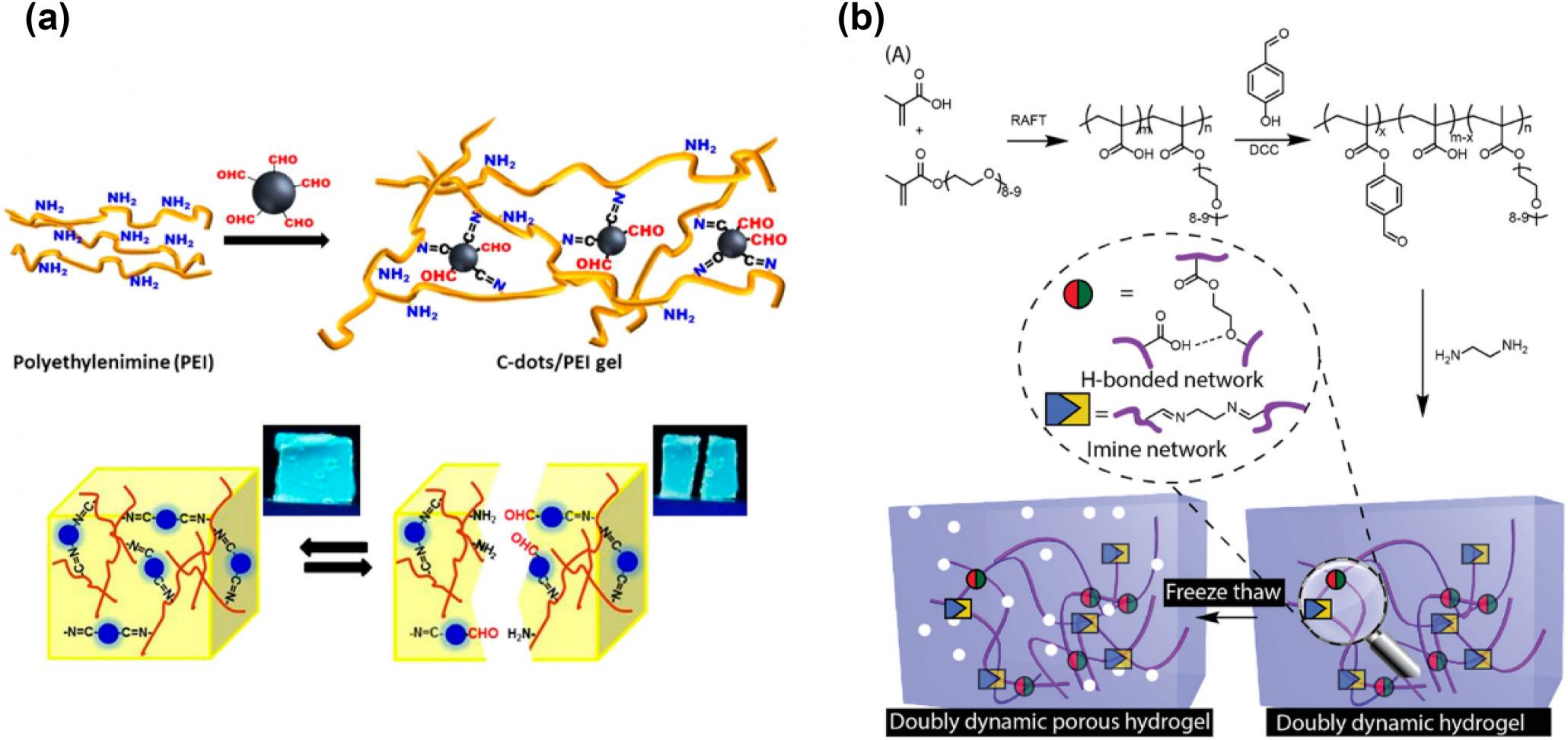
The Schiff‐base rebonding and transamination reaction. (a) Fluorescent self‐healing carbon dot/polymer gels based on Schiff base^[42]^; (b) Self‐healing hydrogels based on imine metathesis and hydrogen bonds.^[44]^ Graph copyright from reference [42, 44].

Schiff base reactions are a kind of catalyst‐free polycondensation in which active carbonyl groups react with amino groups to form imine bonds. In the presence of free primary amino acids, imine metathesis can be realized.[^38^, ^39^, ^40^, ^41^] This reaction has been utilized to create self‐healing gels, such as fluorescent carbon dot/polymer gels.^[42]^ The self‐healing gels were formed through a Schiff base reaction between the aldehyde units displayed upon the carbon dots' surface and primary amine residues within the polyethyleneimine network, generating imine linkages (Figure 2a). The dynamic covalent imine bonds between the carbon dots and polymeric matrix endowed the gels with excellent self‐healing properties and high mechanical strength. The dynamic imine crosslinkages have also been utilized to construct dry ion‐conducting elastomers. However, such elastomers are prone to be damaged or broken in use. To address this issue, a hybrid self‐healing elastomer system combining Schiff‐base and amine reactions was developed. This system exhibits rapid self‐healing, extensibility, and compressibility, making it suitable for wound‐dressing applications. The dynamic reversible imine linkages and hydrogen bonding present in this system enable it to quickly repair any damage or breakage that occurs during use, thereby extending its lifespan and improving its overall performance.^[43]^ Thanks to the dynamic reversible imine linkages and hydrogen bonding, a type of hydrogel with synergistically engineered properties was proposed.^[44]^ The low room temperature glass transition temperature and the rapid kinetic exchange between the imine and hydrogen bonds drive the material to behave in a self‐healed manner (Figure 2b).

#### 2.1.2 S‐S bond

**FIGURE 3:**
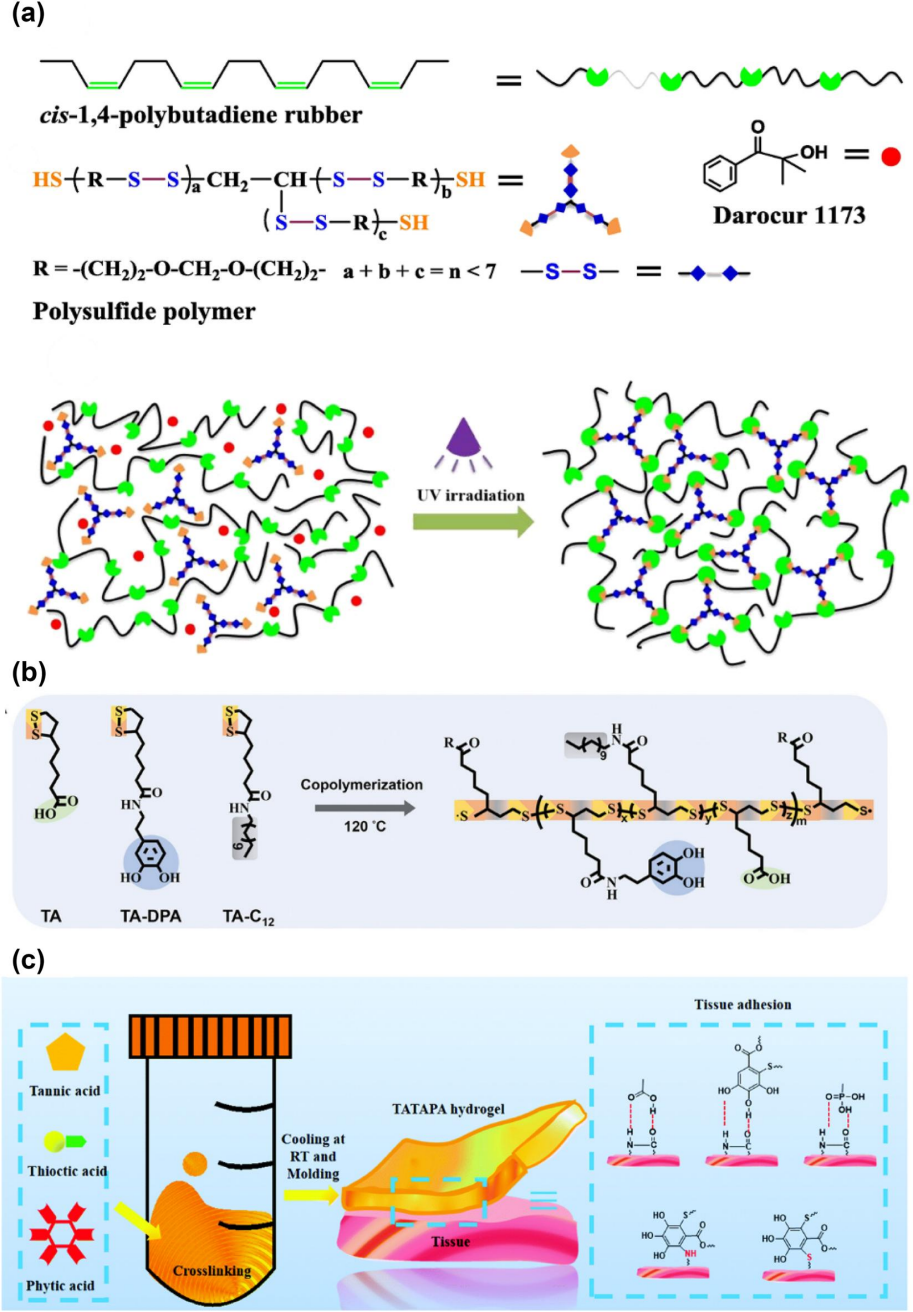
The schematic illustrations of a dynamic disulfide bond. (a) Disulfide metathesis activated by UV irradiation^[16]^; (b) and (c) Schematic illustrations of dynamic disulfide bonds from lipoic acids.[^53^, ^54^] Graph copyright from reference [16, 53, 54].

The S‐S bond energy is reported to be weaker than the C‐C bond energy.^[27]^ This relatively low bond dissociation energy gives the classic disulfide bond an interesting dynamic property.[^45^, ^46^, ^47^] Under UV irradiation or heat conditions, the disulfide bond can be reversibly associated and dissociated. Because of such dynamic features, the disulfide bond has become an essential member of the library of self‐healing building blocks. When the disulfide bonds are embedded in the polymeric backbones, the subsequently constructed materials possess self‐healing and recyclability. Disulfide linkages in polysulfide could dynamically rearrange this crosslinked network through UV‐activated disulfide metathesis (Figure 3a).^[16]^ The healed samples could regain higher than 90% shear strength, and the efficiency was not significantly influenced by the repeated healing process. Due to the dynamic reversibility of disulfide metathesis, the molded rubbers could be transformed into other shapes under UV illumination. Moreover, the pulverized rubber could also be compressed into new items utilizing UV‐induced disulfide metathesis. It is not only for polybutadiene polymers but also for other polymer systems once the introduction of disulfide bonds can impart. After introducing disulfide bonds into a polyester/polyether copolymer,^[48]^ the polymer possessed self‐healing and degradable properties, which can potentially be used in the fabrication of intelligent artificial skin. Conventional epoxy vitrimers are widely used in daily life because of their ability to combine and integrate the advantages of thermoplastics and thermosets. However, the response of a single type of dynamic covalent bond is often slow. Zhang and co‐workers designed a series of epoxy vitrimers containing β‐hydroxyl esters and disulfide bonds. It was significant to combine different types of dynamic covalent bonds within a single polymeric network, each type bearing an adjustable content, to endow these epoxy‐based vitrimers with diversified properties, especially rapid self‐healing abilities.^[19]^

Lipoic acid is a natural small molecule whose five‐membered ring contains a disulfide bond. Under heat or UV irradiation, it is able to undergo ring‐opening polymerization at high concentration due to the negligible ring tension.[^49^, ^50^, ^51^] An unprecedented zwitterionic polyionic elastomer with a dynamic backbone was copolymerized with zwitterionic lipoic acid and other monomers.^[52]^ This dynamer exhibits super stretchability that can be maintained even under stretching, making it a nice candidate to construct smart electronic skins. Besides self‐adhesion and self‐healing, its dynamic feature can afford photo/thermal‐driven remoldability and recyclability. The polymer prepared by hot‐melting lipoic acid had a strong adhesion toward glass, wood, polytetrafluoroethylene and Al.^[53]^ When the side chain of lipoic acid was modified by dopamine (Figure 3b), it can further enhance the adhesion force and be used as an excellent adhesive because catechol groups may interact with different substrates via bidentate H‐bonds, metal–catechol coordination bonds, π–π/π–cation interactions, electrostatic interaction, dipole–dipole and ion–dipole interactions. Due to the dynamical S‐S bond, the adhesive could be reprocessed into a new adhesive so that it reduced the waste of adhesive. In addition to being used in commercial adhesives, lipoic acid‐based materials can also be used in bio‐adhesive materials, such as the TATAPA hydrogel (Figure 3c),^[54]^ which is prepared by three naturally existing organic acids (tannic acid, thioctic acid, and phytic acid) to construct a novel adhesive gel for epidermal tissue bandage applications. These synthetic materials not only maintain existing functions but also gain new self‐healing properties.

In addition to the dynamic covalent bonds mentioned previously, such as the reversible imine and disulfide linkages, other dynamic covalent bonds, such as Diels‐Alder (DA reaction) adducts,[^55^, ^56^, ^57^] boroxines,[^58^, ^59^, ^60^] transesterification reactions,[^61^, ^62^, ^63^] and other types of dynamic covalent bonds, are utilized to construct dynamic self‐healing polymers. These materials not only retain many of the beneficial properties of traditional polymers but also possess a unique ability to undergo repeated healing and recycling due to the dynamic exchange of the dynamic covalent bonds. This dynamic behavior allows the material to maintain its integrity and functionality even in the presence of damage or wear, making it an attractive option for a wide range of applications, from coatings and adhesives to biomedical devices and structural materials.

### 2.2 Dynamic non‐covalent bonds

#### 2.2.1 Coordination bond

**FIGURE 4:**
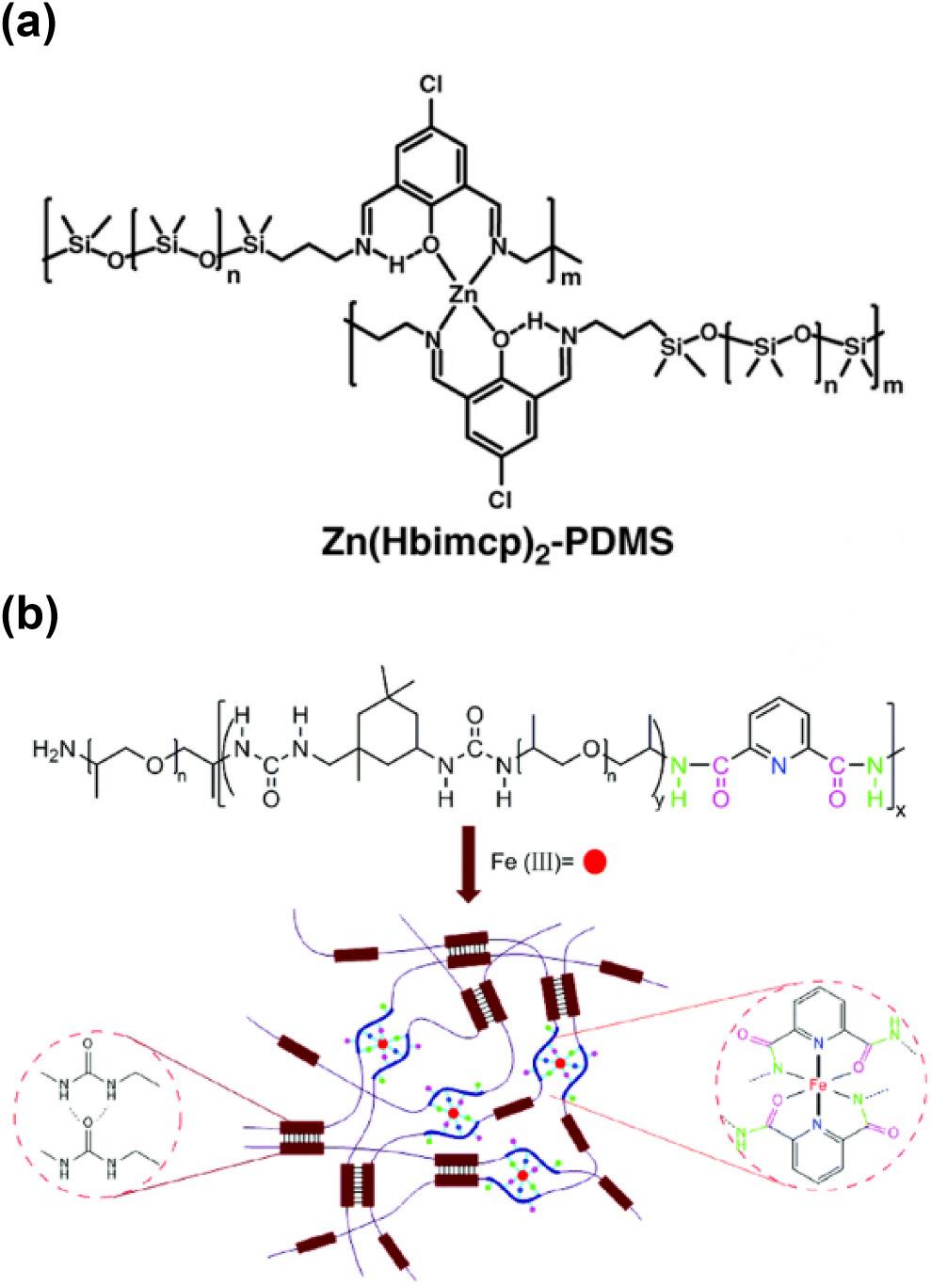
The graphic scheme for metal‐ligand interactions. (a) Dynamic reversible intra‐ and inter‐molecular Zn‐ligand complexes^[67]^; (b) Fe‐ligand (2,6‐ pyridinedicarboxylic acid chloride) complexes.^[68]^ Graph copyright from references [67, 68].

The coordination bonds between metal ions and ligands are a very important type of non‐covalent interaction for achieving self‐healing functions.[^64^, ^65^, ^66^] The metal ions and appropriate ligands could form versatile coordination junctions linking the polymer chains. Different metal‐ligand combinations generally lead to different association strengths. When mechanical forces are applied, the metal‐ligand bonds dissociate, and the subsequent reassociation processes result in bulk self‐healing behavior of the materials. The Zn‐Hbimcp(Hbimcp = 2,6‐bis((imino)methyl)‐4‐chlorophenol) based coordination bond^[67]^ has a relatively large association constant of 2.2 × 10^11^, but can also undergo fast reversible intra‐/inter‐molecular ligand exchange processes (Figure 4a). The as‐prepared Zn(Hbimcp)_2_‐PDMS polymer is highly stretchable with high toughness and could autonomously self‐heal at room temperature. The bond strengths and dynamics of metal‐ligand complexes were believed to be responsible for the material's decent toughness and self‐healing property. The 2,6‐pyridinedicarboxylic acid chloride (Py) could be used as the ligand to coordinate with iron cations (Fe^3+^)^[68]^; the dynamic crosslinking of metal‐ligand polymers resulted in the modulus and elasticity of their supramolecular materials (Figure 4b), showing good transparency, high viscosity, and decent self‐healing performance. The strain of the material could recover within 30 min with its mechanical strength completely recovered. The pyridine group has a lone pair of electrons on its nitrogen atom, which can coordinate with metal ions.[^69^, ^70^, ^71^] Therefore, the pyridine group and metal ions are often used to construct self‐healing supramolecular polymers. The weaker bonds are responsible for energy dissipation on stretching and on‐demand self‐healing, whereas the metal ions maintain their location near the ligands, thus resulting in stronger interactions and rapid bond reformation.

#### 2.2.2 Dipole‐dipole and dipole‐ion interactions

**FIGURE 5:**
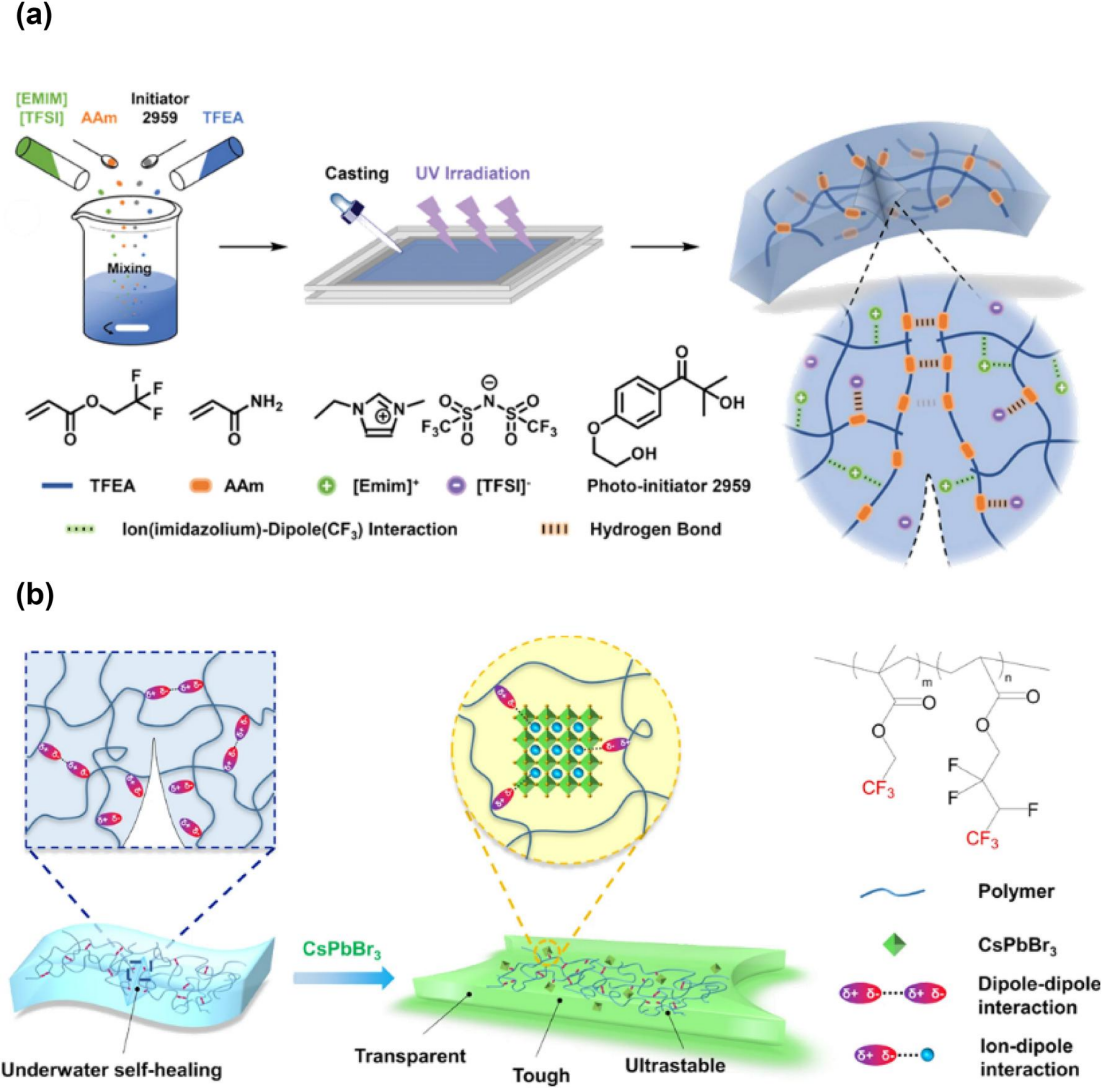
Graphic schemes for dipole‐dipole/dipole‐ion interactions of ‐CF_3_ groups. (a) Graphic illustration of the ion(imidazolium)‐dipole (‐CF_3_) interactions^[74]^; (b) Graphic illustration of the ions in perovskite interacting with CF_3_ to form dipole‐ion interaction.^[75]^ Graph copyright from reference [74, 75].

Dipole‐dipole and dipole‐ion interactions have recently been widely adopted in the molecular design of recently developed materials,[^72^, ^73^] especially trifluoromethyl moieties (‐CF_3_) exhibit strong electronegativity, and two ‐CF_3_ groups can generate strong dipole‐dipole force in between. Meanwhile, the ‐CF_3_ groups can also generate strong electrostatic interactions with ions to form dipole‐ion interactions. These strong non‐covalent interactions can provide materials with excellent mechanical properties. When the dipole‐dipole or dipole‐ion interaction is disrupted, these non‐covalent interactions can be quickly reconstructed at the damage site of the material, which equips the materials with self‐healing properties. Multifunctional ionogels have been reported utilizing abundant non‐covalent interactions, including hydrogen bonding and ion(imidazolium)‐dipole (‐CF_3_) interactions (Figure 5a).^[74]^ These interactions endow ionogels with excellent mechanical strength, decent resilience, and rapid self‐healing capabilities at room temperature. This hydrophobic nature of the ionogels also possesses a unique underwater self‐healing capability due to the fluorine‐rich polymeric matrix bringing in high tolerance against water. Wang and co‐workers proposed a luminescent composite, reported to be highly transparent, tough, and autonomously self‐healing in both dry and wet conditions.^[75]^ The perovskite was doped into fluorine elastomer, and the ions in the perovskite interacted with ‐CF_3_ groups forming strong dipole‐ion interactions (Figure 5b). It increased the degree of crosslinking and therefore toughened the material. The author proposed that this material had excellent self‐healing properties, and the reason for this was that: when the material was damaged, the system shifted from a stable energy state to a relatively unfavorable one. The intensive dipole‐dipole interactions between these highly polar ‐CF_3_ groups at the interface within the polymer network reduced the entropy effect of the network by altering the chain conformations, the broken/separated segments then self‐healed in bulk and the polymer system returned to its initial stable energy state. This reasonable explanation would greatly help researchers to better understand how the ‐CF_3_ group‐induced electrostatic interaction plays a role in this particular self‐healing process. In a word, ionic interactions in polymers provide a unique opportunity for the development of self‐healable commodity materials, and the electrostatic interactions between ‐CF_3_ groups and ions offer the potential for the design of new self‐healing materials with exceptional properties.

#### 2.2.3 Hydrogen bond

**FIGURE 6:**
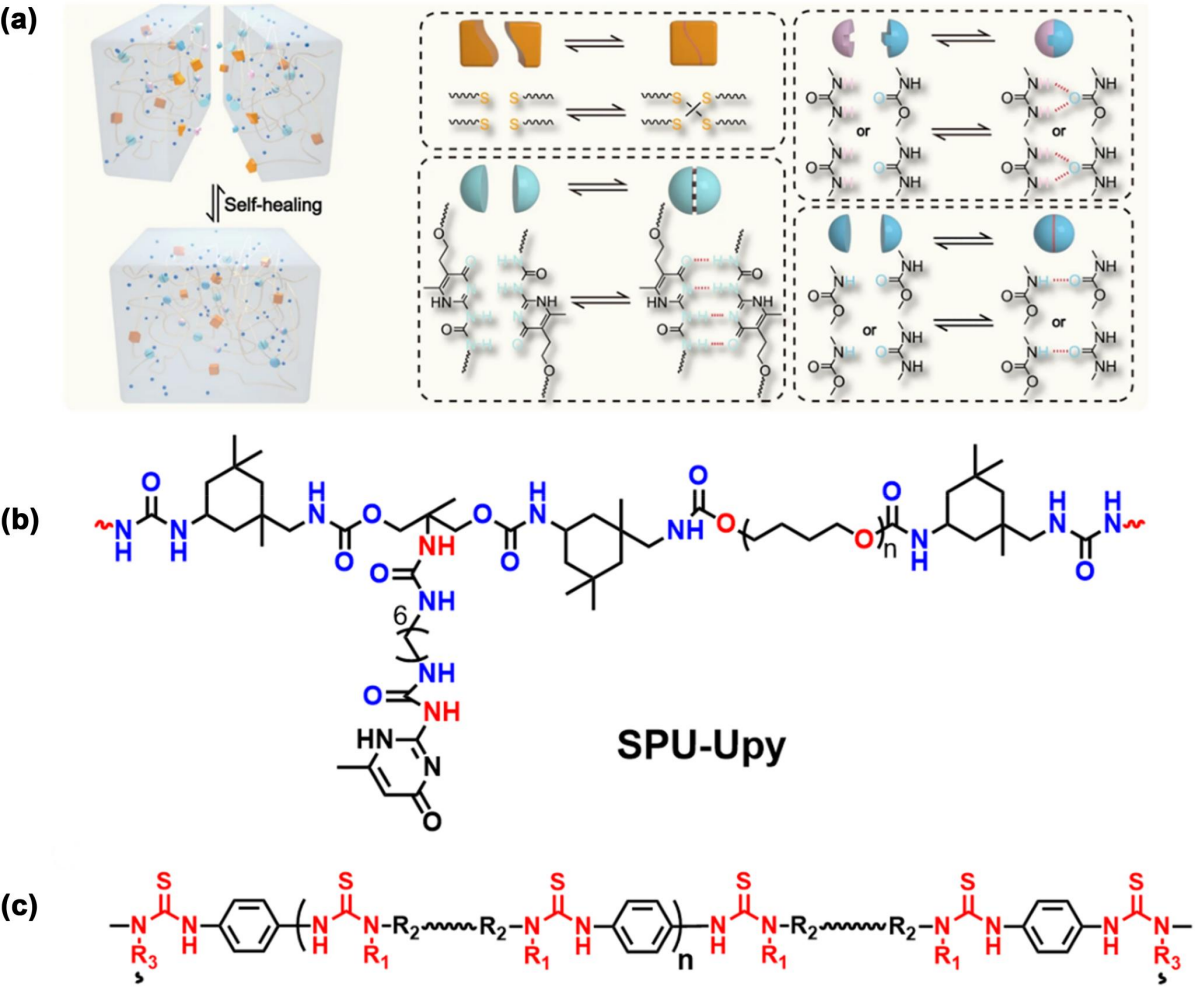
(a) and (b) Graphic schemes of the cooperative crosslinking H‐bonds (UPy‐Upy, urethane‐urethane, urea‐urea, or urea‐urethane)[^81^, ^82^]; (c) Graphic scheme of the cooperative crosslinking thiourea units.^[83]^ Graph copyright from reference [81, 82, 83].

The hydrogen bond is a typical non‐covalent interaction, and its directionality and high per‐volume concentration confer considerable mechanical strength to materials.[^76^, ^77^, ^78^, ^79^, ^80^] Multihydrogen‐bond materials not only have excellent mechanical properties due to strong hydrogen bond interactions within the polymer network but also endowed with self‐healing properties due to the reversibility of the dynamic crosslinking, which is particularly true for polyurethane‐based materials. The dynamic supramolecular ionic conductive elastomers decorated with multiple dynamic interactions, including disulfide metathesis and strong cooperative hydrogen bond crosslinkages (ureidopyrimidinone‐ureidopyrimidinone, UPy‐UPy) and weak anti‐cooperative hydrogen bond crosslinkages (urethane‐urethane, urea‐urea, or urea‐urethane) exhibit excellent self‐healing capability at room temperature and favorable recyclability due to the synergistic cooperation of different dynamic bonds (Figure 6a).^[81]^ The synergistic cooperation of the different dynamic bonds contributed to the excellent self‐healing capability (∼99% at room temperature) and the favorable recyclability. The 2‐ureido‐4‐pyrimidone (UPy) group can be used as a dangling chain to coordinate with additional zinc ions, further increasing the amount of crosslinkages within the network and strengthening the mechanical properties of the material (Figure 6b).^[82]^ At the same time, this material handed an unexpected high self‐healing efficiency of 95% at ambient temperature within 24 h. Zhang and co‐workers developed a type of catalyst‐free reversible polythiourea directly built from commodity 1, four‐ phenylene diisothiocyanate and amines via the facile click chemistry‐based molecular assembly (Figure 6b).^[83]^ Using the amine motifs with various steric hindrances and flexibilities, the dynamic reversible thiourea units acquired triggering temperatures from room temperature to 120°C. Accordingly, these self‐healable dynamic crosslinked polythioureas, which can be degraded and recycled in a controlled manner, could potentially functionalize in a wide temperature range.

Although the self‐healing performance of these materials discussed here is similar, the utilization of dynamic motifs and mechanisms of dynamic transformation processes are quite distinguishing. Different external stimuli and exchange mechanisms act together to activate the self‐healing processes. Each kind of dynamic interaction and different dynamic molecular motifs have their advantages and disadvantages. For example, although the bond energy of disulfide is low, the dynamic exchange of disulfide bonds can be realized under thermal stimulation with excellent self‐healing performance. Under certain conditions, disulfide bonds are prone to undergo oxidation reactions, resulting in complete loss of their dynamic properties. Although hydrogen bonds are considered as weak interactions, they can rapidly exchange at lower temperatures. Therefore, when using molecular motifs with intrinsic dynamicity as self‐healing building blocks, we should fully balance the nature of materials between mechanical properties and self‐healing performances. As far as possible, such synthetic materials have excellent mechanical properties while still having excellent self‐healing properties.

## 3 SHAPE‐SHIFTING MATERIALS

When the natural environment changes, creatures usually adjust or modify their shape to adapt to the changes in the environment through physical and chemical cooperation actions. The basis for the occurrence of physical and chemical interactions is stimuli‐responsive motifs that could achieve macroscopic shape changes. To simulate these shape transformation phenomena, dynamic chemical bonds and dynamic reversible molecular motifs are undoubtedly the best candidates as actuating units to drive shape‐shifting materials because they have the ability to respond to all kinds of stimuli. When they are embedded in the polymer network in a controlled manner, the dynamic bonds and molecules in the polymer matrix will change dynamically under the induction of external stimuli,[^84^, ^85^, ^86^] and the molecular force could be amplified step by step, finally resulting in a change of the macro‐shape of the polymer. The successful preparation of these smart materials cannot be achieved without an elaborate material design. On the one hand, stimuli‐responsive groups, loading polymer matrix and stimulation methods should be properly selected. On the other hand, the physical forms of material (such as hydrogels, organogels and dry network polymers) are also important, different forms have different actuated environments. The obtained shape‐shifting materials could result in stimulus‐responsive behavior in some scenarios that can mimic the performance of organic organs to some extent.

### 3.1 Evolution from solid polymers to hollow‐structure polymers

**FIGURE 7:**
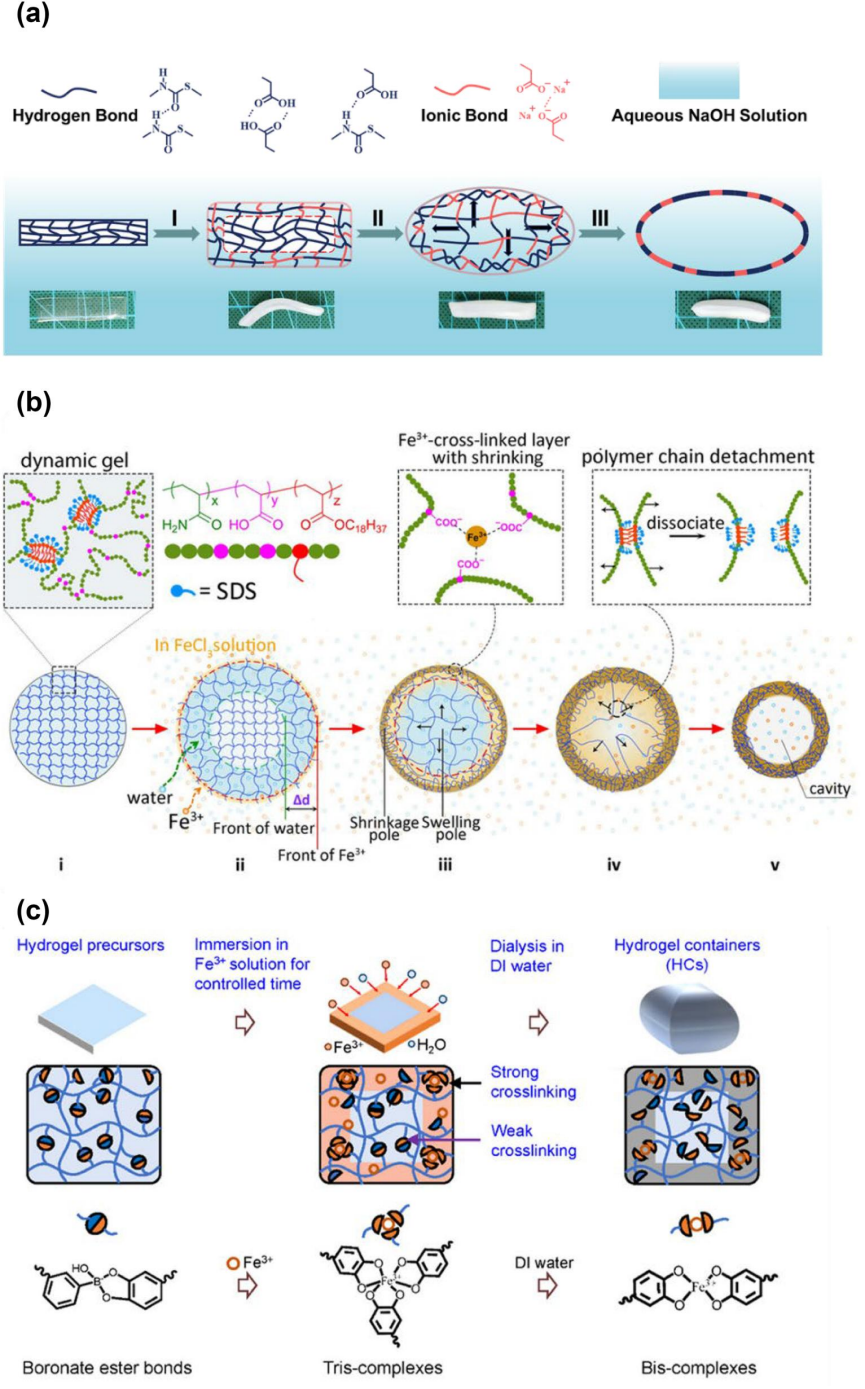
(a) Schematic illustration of shape‐shifting behavior induced by the transformation from hydrogen bonds to ionic bonds^[90]^; (b) Creation of a field with “a swelling pole” and “a shrinking pole” to realize a self‐evolution^[91]^; (c) Coordination‐driven network contraction.^[92]^ Graph copyright from reference [90, 91, 92].

Hollow structures are essential for many functions.[^87^, ^88^, ^89^] However, it is still challenging to realize macroscopic shape‐evaluation‐like transitions from solid to closed hollow structures. Recent examples from Qu and Cui's groups demonstrate self‐evolving polymers and hydrogels that undergo macroscopic shape‐shifting to form neat hollow structures. Qu and co‐workers' recent example demonstrated a self‐evolution polymer (Figure 7a) from a polythiourethane‐based dry network.^[90]^ The virgin film was crosslinked by intensive hydrogen bonds between the thiourethane backbones and carboxyl of dangling chains. The transition of the microscopic molecules induced a macroscopic shape‐shifting of the polymer, and the degree of polymer shape transition could be regulated by immersion time and concentration of Fe^3+^ and NaOH. Cui and co‐workers introduced the terms “a swelling pole” and “a shrinking pole” to realize a self‐evolution strategy (Figure 7b)^[91]^; these dynamic conditions drove the polymers to disassemble, migrate, and resettle in the targeted region. The synergistic effect of the conversion of hydrogen bonding to coordination interactions, and the transition of hydrophilic interactions to hydrophobic ones, induced the macroscopic hollow structures of the studied hydrogels. With a similar mechanism, an in‐situ method was used in a direct spatial surface‐interior separation from the bulk dynamic hydrogels to the closed three‐dimensional hydrogel containers with inner cavities by constructing a cross‐linking gradient within the dynamic hydrogels.^[92]^ The initial cross‐linking of phenylboronic acid/catechol complexes was disrupted by stronger ferric ions/catechol associations (Figure 7c), which gradually weakened the cross‐linking from outside to inside. Both the stronger cross‐linking in the outer shells and sequentially weaker cross‐linked interiors were generated during the swelling process of the closed hydrogel container with a tunable dense outer shell, fluffy inner layer and cavities in the core. The role of coordination is believed to be the driving force for the material's shape‐shifting. Before and after coordinating with the ion, the inner stress coming from the polymer network is different; the difference in internal stress induced the material's shape changes.[^93^, ^94^, ^95^, ^96^] The driving force for the material's shape‐shifting is believed to be the role of coordination, where the difference in internal stress induced by the coordination of the ion before and after coordination with the polymer network results in the material's shape changes. One interesting example demonstrated a light‐driven hydrogel actuator with a hollow sphere that could jump (height: 15 cm) and roll in the air with high speed and fast response.^[17]^ The mechanism of the jumping behavior was that: the rapid temperature increase originated from the photothermal effect of IONPs, causing the gasification of water and bubble formation. The gas bubble hit the area of light radiation, causing the shell to deform and expand. The fast‐expanding local protrusion hit the substrate, and the counter interaction provided the driving force for the jumping behavior.

### 3.2 Liquid crystal (LC) actuator

**FIGURE 8:**
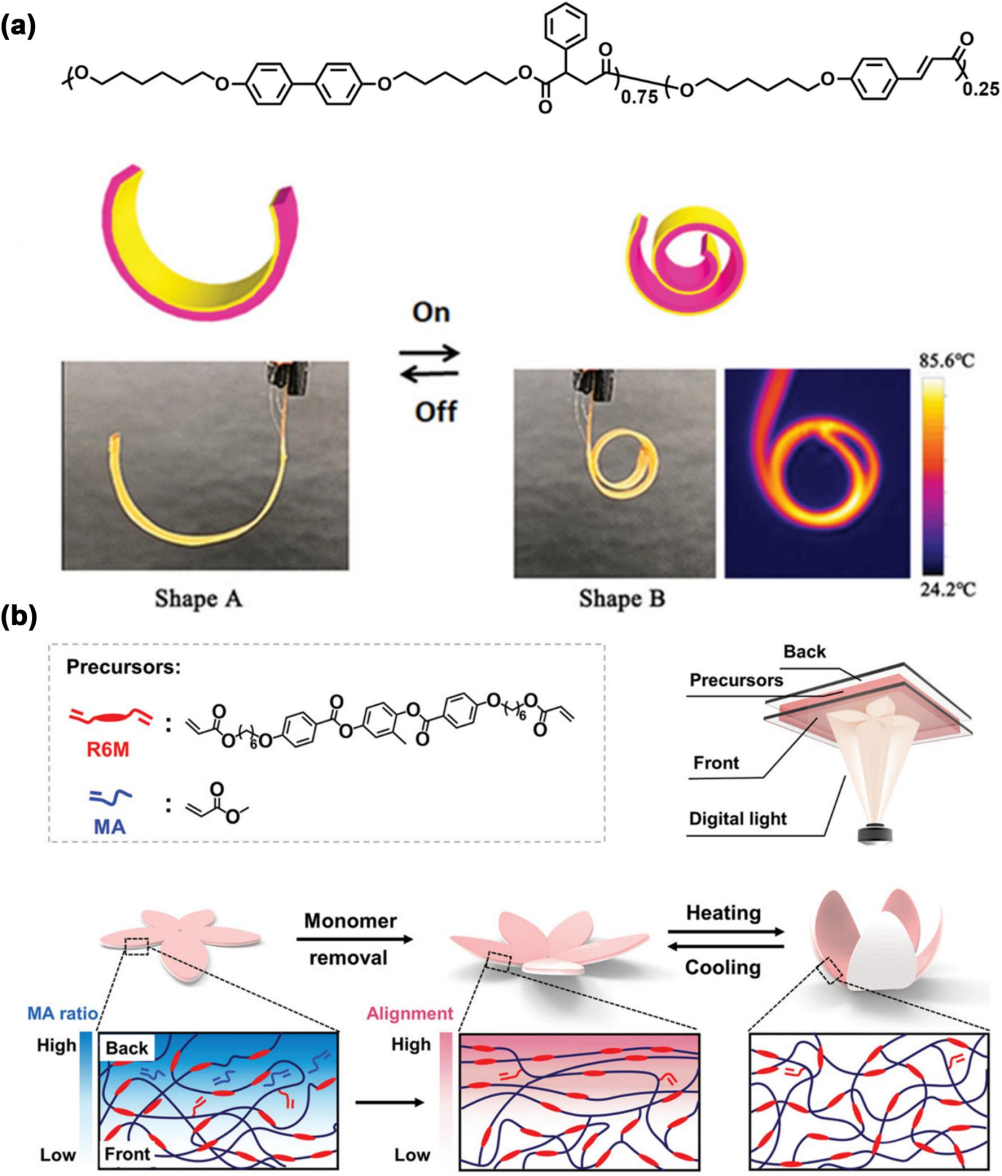
(a) Graphic illustrations of the joule heating effect‐actuated bilayer actuator of a LCN/polyimide^[18]^; (b) The actuator of the liquid crystal alignment gradient effect.^[110]^ Graph copyright from reference [18, 110]

Liquid crystal (LC) is well known as an excellent stimulus‐response element for actuators.[^97^, ^98^, ^99^, ^100^] Under external stimuli, such as temperature changes or the application of electric fields, the deformations induced by phase transitions or realignment of LCs are notably rapid. These stimuli prompt swift alterations in the LC structure and alignment, resulting in substantial deformations on a macroscopic scale. This can exhibit the reversible, complex, and colossal amplitude shape deformation along with the external‐stimuli‐triggered order‐disorder phase transition of the internal mesogenic polymer network. Such versatile LCs have a prosperous application prospect in intelligent actuators,[^101^, ^102^, ^103^] robotic technology,[^104^, ^105^, ^106^] and biomedical engineering.[^107^, ^108^, ^109^] The concept of a “Janus” soft robot actuator network based on integrated oriented LC networks (LCNs) was demonstrated (Figure 8a) by building a bilayer actuator of an LCN and polyimide (Kapton).^[18]^ The heating wire was physically embedded in the LC polymer; the Joule heating effect was used to induce actuation response; the heat supply can be located inside the material and controlled in real‐time due to the highly controllable electrical stimuli in terms of amplitude, duration and phase. Xie and co‐workers proposed a digital photocuring method for ultrafast template‐free fabrication of LCE artificial muscles capable of designable complex motions (Figure 8b).^[110]^ A gradient in the degree of UV light curing leads to a LC alignment gradient from the front to the back. Under thermal stimulation, the polymer film would also have a difference in intrinsic force that creates mesogen alignment for reversible bending. The LCs are excellent stimulus‐response elements for actuators, and their versatile properties have a prosperous application prospect in various fields.

### 3.3 Photo‐responsive polymers

**FIGURE 9:**
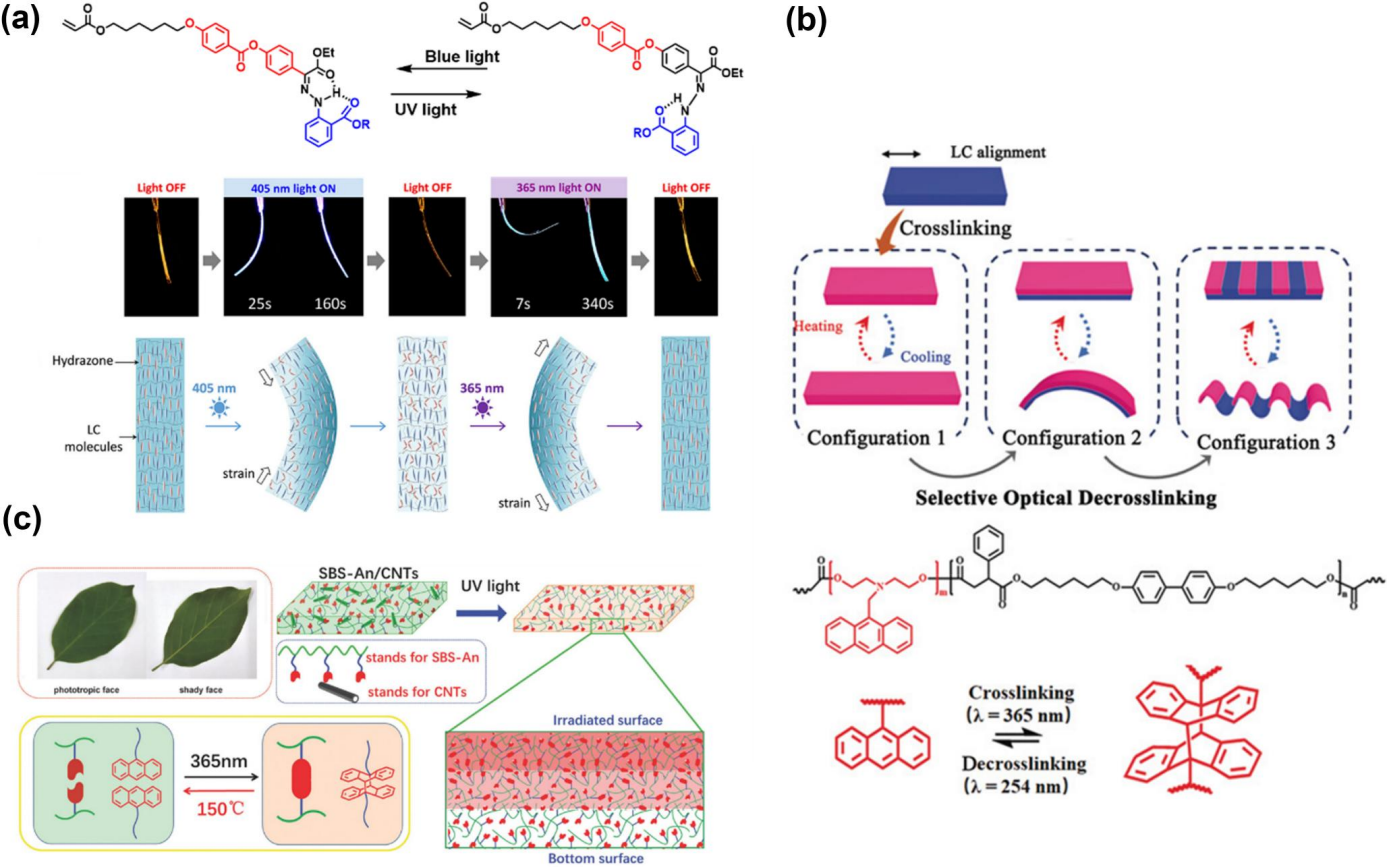
(a) Graphic illustrations of a liquid crystal actuator with hydrazone photoswitch^[15]^; (b) and (c) The Janus structure actuator of bilayers comprised different gradients on the dimerization of anthracene.[^114^, ^115^] Graph copyright from references [15, 114, 115].

Light, spatiotemporally controlled and much more accessible than any other stimuli, enables non‐invasive and remote manipulation of hydrogel actuators with excellent spatial and temporal resolution. Photo‐responsive molecules are functional blocks that can photoisomerize, photodimerize and cleavage/reconstruct bonds under external light stimulation.[^111^, ^112^, ^113^] When these blocks are introduced into the polymer network, it becomes a light‐responsive polymer, which can be actuated by the external light resources. A hydrazone photoswitch was integrated with the cooperation of the LC to yield a polymer network (Figure 9a) that responded to the light with significant shape transformations.^[15]^ When this crosslinker of switch monomer was incorporated covalently with a photopolymerizable LC, a significant shape transformation was observed under external illuminations. This observation introduced a shape‐shifting mechanism based on photogenerated stresses because only these cross‐linked molecular switches could induce such a shape transformation by photoisomerization.

The anthracene molecule motif is best known for its reversible photodimerization and photocleavage of dimers under UV irradiation at two different wavelengths or by heat. Zhao and co‐workers prepared an anthracene‐containing LC polymer network (LCN)^[114]^; the dimerization of anthracene led to the cross‐linkage formation of the polymer network (Figure 9b). The LC actuator displayed reversible contraction (in the disordered isotropic phase) and extension (in the ordered LC phase) along the initial LC orientation direction. The non‐crosslinked monodomain sample showed negligible reversible shape change, which was an indication of the anthracene dimerization. This crosslink dramatically enhanced the deformation magnitude of the actuator. Also, the anthracene dimer‐based crosslink points could be dedimerized by thermal effects.^[115]^ The elastic matrix of the Janus structure (bilayers comprised of different gradients) was doped with carbon nanotubes (CNTs) and photoreactive anthracene (Figure 9c). When the polymer was exposed to UV light, the degree of anthracene dimerization gradually increased from bottom to top because of the efficient CNT photothermal energy transformation. The solvent or shape memory effect induced shape morphing was obtained via the heterogeneous swelling (deswelling) ability of internal stress from the Janus structure. The anthracene‐containing LC polymer networks show promising potential as stimuli‐responsive actuator materials. By carefully controlling the crosslinking process and incorporating other elements, these materials can exhibit a range of complex and reversible shape changes in response to external stimuli, including heat and light.

Unlike self‐healing materials, shape‐shifting materials require more sophisticated actuation units and polymer matrixes. When the actuation units of dynamic bonds/molecules respond to external stimuli, these units can amplify the dynamic reversible effect of the molecular unit step by step, and subsequently drive the macroshape transformation of the material. This process requires the incorporation of dynamic molecules and the assistance of a polymer network. Incorporation of dynamic bonds/molecules into the polymer network can be achieved through various methods, including covalent or non‐covalent crosslinking, physical blending, and co‐assembly. The design of the actuation units and the polymer network structure can be optimized to achieve the desired shape‐shifting properties, such as the magnitude and direction of shape change, response time, and stability. The combination of different actuation units and polymer network structures can lead to the development of advanced shape‐shifting materials with a wide range of potential applications, including soft robotics, biomedical engineering and intelligent materials.

## 4 COMBINATION OF SELF‐HEALING AND SHAPE‐SHIFTING MATERIALS

Materials that possess combined self‐healing and shape‐shifting capabilities have garnered significant attention and research interest. These materials exhibit unique properties that enable them to autonomously repair damage and alter their shape, presenting tremendous potential for various applications.[^116^, ^117^, ^118^, ^119^, ^120^, ^121^] Despite notable advancements in materials with combined self‐healing and shape‐shifting capabilities, challenges and opportunities persist. These include the synthesis methods of materials, investigations into self‐healing and shape‐shifting mechanisms, material stability, and controllability. Through in‐depth research and innovation, further enhancement of properties of these materials and expansion of their application domains can be achieved, enabling the creation of more intelligent and sustainable material solutions.

## 5 SUMMARY AND OUTLOOK

In this review, we have summarized self‐healing and shape‐shifting polymers based on dynamic reversible interactions. These materials have shown great potential in various applications, including soft robotics, biomedical engineering, and intelligent materials.

However, there are still many challenges and deficiencies that need to be addressed in the development of dynamic materials. For example, synthesizing certain dynamic molecules can be complicated and tedious, which limits their applications in advanced material fabrication. Improving the synthesis route and yield is an effective way to solve this problem. Additionally, the response speed of dynamers embedded in the polymer network can be slow due to various factors, and low energy conversion efficiency is also a big obstacle for stimuli‐responsive materials. Therefore, the design of materials must be ingenious to achieve rapid responses as soon as possible, and accurately controlled in terms of spatial and time dimensions.

Furthermore, the ultimate purpose of developing updated functional materials is to better facilitate our daily lives, which requires materials to have both economy and practical functions, such as a simple and straightforward preparation process, lower cost, faster self‐healing and shape‐shifting speed, and higher mechanical strength. It will require scientists to spend more time and energy to innovate and explore the engineering feasibility of materials.

In conclusion, the development of dynamic materials based on dynamic chemistry is an exciting field with great potential. There is still ample room for scientists to innovate and explore new dynamic bonds/molecules and new stimulation methods to achieve a better understanding of these dynamic building blocks. The future of intelligent materials based on dynamic chemistry is bright and prosperous, with many opportunities for further advancements and discoveries.

## AUTHOR CONTRIBUTIONS

The manuscript was written through the contributions of all authors. All authors have given approval to the final version of the manuscript.

## CONFLICT OF INTEREST STATEMENT

The authors declare no conflicts of interest.

## Data Availability

The data that support the findings of this study are openly available.
